# Multi-Antigen Elephant Endotheliotropic Herpesvirus (EEHV) mRNA Vaccine Induces Humoral and Cell-Mediated Responses in Mice

**DOI:** 10.3390/vaccines12121429

**Published:** 2024-12-18

**Authors:** Jessica R. Watts, Jennifer L. Spencer Clinton, Jeroen Pollet, Rongsheng Peng, Jie Tan, Paul D. Ling

**Affiliations:** 1 Department of Molecular Virology and Microbiology, Baylor College of Medicine, Houston, TX 77030, USA; jwatts@bcm.edu (J.R.W.); rpeng@bcm.edu (R.P.); jtan@bcm.edu (J.T.); 2 Department of Pediatrics, Division of Tropical Medicine, Baylor College of Medicine, Houston, TX 77030, USA; jennifer.clinton@bcm.edu (J.L.S.C.); jeroen.pollet@bcm.edu (J.P.); 3 National School of Tropical Medicine, Baylor College of Medicine, Houston, TX 77030, USA

**Keywords:** elephant endotheliotropic herpesvirus, mRNA vaccines, multivalent vaccine

## Abstract

**Background/Objectives:** Elephant endotheliotropic herpesvirus (EEHV) causes lethal hemorrhagic disease (HD) in Asian and African elephants in human care and the wild. It is the leading cause of death for young Asian elephants in North American and European zoos despite sensitive diagnostic tests and improved treatments. Thus, there is a critical need to develop an effective vaccine to prevent severe illness and reduce mortality from EEHV-HD. We generated a multi-antigenic EEHV mRNA vaccine to address this need that encodes the EEHV1A-subtype glycoproteins gB, gH, gL, and gO. These conserved proteins are the entry machinery for several herpesviruses in the betaherpesvirus subfamily and elicit humoral and cellular immunity in naturally infected elephants. **Methods:** Outbred CD-1 mice were vaccinated with two doses of an mRNA vaccine comprising modified EEHV1A gB, gH, gL, and gO mRNAs encapsulated into lipid nanoparticles. Humoral and T-cell immunity was assessed three weeks after the first dose or three weeks after the booster dose using luciferase immunoprecipitation system assays and flow cytometry, respectively. **Results:** The CD-1 mice vaccinated once had detectable antibody titers against gB, gH, and gL that increased significantly three weeks after a booster dose. Activated CD4^+^ and CD8^+^ T cells secreting cytokines associated with a T_H_1 response were induced against all four glycoproteins. No adverse effects were observed following one or two doses of the vaccine. **Conclusions:** We found that gB, gH, gL, and gO as a multivalent vaccine stimulated robust humoral and cell-mediated immunity. This is a critical step for moving this candidate EEHV1A mRNA vaccine into clinical trials in Asian elephants.

## 1. Introduction

Elephant endotheliotropic herpesvirus (EEHV) causes lethal hemorrhagic disease (HD) in Asian (*Elephas maximus*) and African Savannah (*Loxodonta africana*) elephants [1]. EEHV-HD is the leading cause of mortality for juvenile Asian elephants in North America and Europe, and over 100 cases of EEHV-HD have been identified among Asian elephants in semi-captive and wild populations in natural range countries located in various parts of Asia [1]. To our knowledge, there has not been a comprehensive analysis of the prevalence of EEHV incidents in free-roaming Asian elephants. Although concerns have been focused largely on EEHV-HD in Asian elephants, increased cases in African Savannah elephants in North American institutions have raised concerns about EEHV in African elephants [2,3]. EEHV prevalence or disease in African Forest elephants (*Loxodonta cyclotis)* remains unknown.

Seven EEHV types in the subfamily *Betaherpesviridae* and the genus *Proboscivirus* circulate in Asian and African elephants and likely co-evolved separately with these elephant species [4]. EEHVs 1, 4, and 5 are endemic to Asian elephants, whereas EEHVs 2, 3, 6, and 7 circulate in African elephant populations [1,5,6]. Each major EEHV type includes genetically distinct subtypes (e.g., 1A, 1B, 4A, 4B, etc.) [1,7,8,9]. Although most adult elephants are chronically infected with one or more EEHV types, EEHV-HD is associated with the EEHV1A and 1B subtypes in Asian elephants [1,2] and with EEHVs 2 (Erin Latimer, personal communication) and 3 [10] in African elephants. Presently, it is unknown whether prior infection with other EEHV types provides any sort of cross-protection against another type. Therefore, our initial efforts are focused on generating an effective vaccine against the 1A subtype since it is the subtype that is associated with the vast majority of EEHV-HD cases. The rapid onset of EEHV-HD with few or no symptoms can delay treatment, including plasma transfusions, intravenous fluids, and human anti-herpesvirus drugs [11,12,13]. However, in some cases, these interventions have been insufficient to prevent mortality.

Most herpesvirus infections are either benign or cause only minor self-limiting diseases. The factors contributing to lethal EEHV infection in elephants are not well understood, but a reduction in or loss of maternal antibodies is associated with vulnerability to a lethal primary infection. Elephant dams passively transfer EEHV-specific antibodies to calves at birth, which decrease at 1–3 years of age, when elephants are most susceptible to EEHV-HD [14,15]. In both Asian and African elephants, the loss of maternal antibodies leaves calves susceptible to serious disease following a primary EEHV infection [10,14,16].

The trimer complex of glycoproteins H, L, and O (gH/gL/gO) and the viral fusion glycoprotein B (gB) serve as the conserved herpesvirus entry machinery [17]. In healthy human adults, cellular and humoral immunity against gB and the trimer complex play important roles in conferring and maintaining protection from herpesvirus infection [18,19]. Several herpesvirus vaccines in development that incorporate one or more of these conserved glycoproteins induced robust immune responses in several model systems [20,21,22]. EEHV sequencing and serology data show that these glycoproteins are made and are highly immunogenic in naturally infected elephants [14,23,24]. We also showed that adult elephants that are naturally infected with EEHV generate antibodies against the conserved herpesvirus machinery [14], and gB has been identified as a specific target for cell-mediated immunity in chronically infected elephants [25].

EEHV vaccine developments have been hampered by a lack of in vitro EEHV culture systems. Therefore, traditional vaccines, such as live attenuated virus vaccines, are not feasible [1,26]. Our lab has generated prototype EEHV vaccines including a gB-modified vaccinia Ankara (MVA) recombinant virus vaccine [27] and adjuvanted protein subunit vaccines for gB [28] and the gH/gL complex [29]. While these vaccines have been shown to induce both a humoral and cell-mediated immune response, there are concerns over its adjuvant safety in elephants and the regulatory measures. Therefore, additional vaccine platforms that can accommodate multiple antigens in a formulation with lower reactogenicity have been identified. mRNA vaccines activate the adaptive immune response and subsequently induce potent humoral and cytotoxic T-cell responses for a variety of pathogens [30,31]. There are mRNA vaccines for human herpesviruses, including Epstein–Barr virus (EBV), human cytomegalovirus (HCMV), herpes simplex virus (HSV), and varicella zoster virus (VZV), that include one or more glycoproteins and elicit strong, durable antibody and cellular responses in animal models, including mice and nonhuman primates [32,33,34,35,36,37]. We demonstrated previously that a gB recombinant modified vaccinia Ankara (MVA) vaccine and a gB or gH/gL adjuvanted subunit vaccine induced robust antibody and polyfunctional T-cell responses in vaccinated mice [27,28,29]. Since our ability to test single versus multi-antigen vaccine formulations in elephants is limited, we postulate that a multi-antigen EEHV1A vaccine incorporating glycoproteins gB, gH, gL, and gO will give us the best chance of inducing a robust humoral and cell-mediated immune response that protects juvenile elephants from a lethal EEHV1A primary infection. In this study, we evaluate the ability of an EEHV1A multi-antigen gB, gH, gL, and gO mRNA vaccine to induce robust immunity with no adverse effects in mice. This is an important step in moving this candidate vaccine into trials in elephants.

## 2. Materials and Methods

### 2.1. Generation of EEHV1A mRNA Transcripts and Lipid Nanoparticle (LNP) Encapsulation

**Figure 1:**
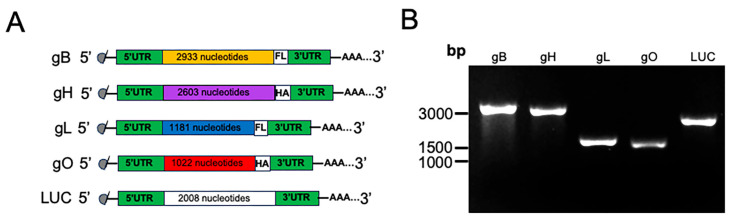
Characterization of in vitro-transcribed mRNA integrity. (**A**) Schematic of mRNA transcripts. Created in BioRender.com. Carboxy-terminal HA and FLAG(FL) protein epitope tags are indicated in the diagram. (**B**) mRNA transcripts visualized on a 1% native agarose gel. Luciferase mRNA (LUC) was included as the control for the vaccine study.

Codon-optimized cDNA sequences for gB (2933 bp), gH (2603 bp), gL (1181 bp), and gO (1022 bp) derived from the EEHV1A Kimba strain, a prototype EEHV1A strain (GenBank: KC618527), were synthesized by GenScript (Piscataway, NJ, USA). Glycoproteins gB and gL were engineered to express carboxy-terminal FLAG epitope tags, whereas gH and gO express carboxy-terminal HA epitope tags. The native signal sequences of each glycoprotein, along with a Kozak sequence and identical 5′ and 3′ human UTR sequences, were included for increased mRNA stability and translation, as shown in Figure 1A. The control luciferase mRNA was designed similarly. In vitro mRNA transcripts were made using T7 polymerase and capped using TriLink CleanCap technology (San Diego, CA, USA) from a linear DNA template from the Houston Methodist Research Institute’s RNAcore (Houston, TX, USA), as described previously [38,39]. The final mRNA transcripts included a 5′ cap, 5′ and 3′ UTRs, 100% N1-methylpseudouridine-5′-triphosphate (m^1^ΨTP) base modification, and a 150-nucleotide poly-A tail (Figure 1A). We used qualitative gel electrophoresis to assess the purity and integrity of the mRNA transcripts (Figure 1B). Control luciferase mRNA transcripts were made using the same procedures outlined previously for the EEHV mRNAs. In vitro mRNAs were formulated into LNPs with the GenVoy-ILM lipid reagent (Precision NanoSystems, Vancouver, BC, Canada) as described previously [40,41]. Briefly, the lipid mixture was dissolved at 12.5 mM in ethanol at a molar ratio of 50:10:38:1.5 (DOTAP:DSPC:cholesterol:PEG-DMG). mRNAs in an aqueous solution were diluted in 50 mM sodium acetate buffer (PNI Formulation Buffer, Precision NanoSystems, pH 4.0) to a working concentration of 0.17 μg/mL and mixed with lipids at a ratio of 3:1 using a benchtop Precision NanoSystems microfluidic mixer (Vancouver, BC, Canada). The LNPs were diluted with 1× phosphate-buffered saline (PBS) with no magnesium or calcium (Corning, Corning, NY, USA) and dialyzed through centrifugation using EMD Millipore Amicon Ultra Centrifugal Filters (Burlington, MA, USA). The particle size, encapsulation efficiency, and pKa were determined using a Zetasizer dynamic light scattering reader (London, EN, UK), an Invitrogen RiboGreen RNA Assay Kit (Waltham, MA, USA), and a 6-p-toluidinylnaphthalene-2-sulfonate (TNS) fluorescence assay, respectively. The formulations were concentrated in 8% sucrose and filtered through 0.22 μm filters for storage at −80 °C for up to 6 months. Immediately before vaccination, 2.5 μg of each EEHV1A mRNA + LNP formulation was defrosted on ice and mixed to prepare a 10 μg vaccine with a total volume of 50 μL. As a control, 10 μg of luciferase + LNPs at a total volume of 50 μL was defrosted and administered to the control mice.

### 2.2. Immunoblot

We transfected 2 × 10^5^ human embryonic kidney cells (HEK293T) purchased from ATCC (Manassas, VA, USA) with 2.5 μg of either gB, gH, gL, or gO mRNA using the Millipore Sigma Ribojuice mRNA Transfection Kit (Burlington, MA, USA). An mRNA transcript encoding green fluorescent protein (GFP) (L-7601, Trilink) was used as a positive control for the transfection experiment. After 24 h, the cells were harvested with 1× PBS and centrifuged at 3000 RPM for 3 min. The cell pellet was resuspended in 2× lysis buffer (100 mM Tris pH 6.8, 4% sodium dodecyl sulfate [SDS], 20% glycerol, 3% beta-mercaptoethanol, and 5% bromophenol blue) and sonicated for 10 s. Then, samples were boiled at 95 °C for 5–10 min and placed on ice for 3 min before centrifugation at 15,000 RPM for 5 min. The samples and the Thermo Scientific PageRuler Prestained Protein Ladder (Waltham, MA, USA) were loaded onto 8–10% SDS polyacrylamide gel, and the proteins were separated using electrophoresis and then transferred onto a nitrocellulose membrane at 20 V for 30 min using the Bio-Rad Trans-Blot Turbo System (Hercules, CA, USA). Following the transfer, the membranes were washed with 1× PBS and incubated with 5% blocking buffer (dry nonfat milk in 1× PBS) for 5 min. The immunoblots were probed with either a Biolegend (San Diego, CA, USA) monoclonal mouse anti-HA antibody (Clone16B12, 1:1000 dilution) or a Sigma Aldrich (St. Louis, MO, USA) monoclonal mouse anti-FLAG antibody (Clone F3165, 1:1000 dilution) in 0.5% blocking buffer overnight with continuous rocking at 4 °C. The immunoblots were incubated with a peroxidase-conjugated Jackson ImmunoResearch (West Grove, PA, USA) secondary goat anti-mouse IgG antibody (1:2000 dilution) at room temperature (RT) for 30 min, developed with Thermo Fisher Scientific Super Signal West Pico Chemiluminescent Substrate (Waltham, MA, USA) and imaged on an Azure Biosystems 300Q chemiluminescent imaging system (Dublin, CA, USA).

### 2.3. Immunofluorescence Staining

We transfected 5 × 10^5^ HEK293T cells separately with each of the mRNAs for 24 h and then trypsinized and seeded 5 × 10^4^ cells onto coverslips in 24-well plates pre-treated with 0.1 mg/mL Cultrex poly-D-lysine (Minneapolis, MN, USA) for 2 h. Treated wells were washed with 1× PBS and incubated at 37 °C overnight with complete DMEM (GenDEPOT, Baker, TX, USA). The cells were fixed with 4% formaldehyde/PEM buffer (0.5 M EGTA pH 7.0, 80 mM dipotassium PIPES pH 6.8, 2 mM MgCl_2_) for 30 min at RT and washed with PEM before permeabilizing them with PEM + 0.5% Tween 20 for 30 min. The cells were blocked with 5% blocking buffer (dry nonfat milk in TBS +0.1% Tween 20) before incubating them with either a monoclonal mouse anti-HA antibody (Biolegend, Clone 16B12, 1:500 dilution) or a monoclonal mouse anti-FLAG antibody (Sigma Aldrich, Clone F3165, 1:500 dilution) for 1 h at RT. The cells were subsequently incubated with an Invitrogen (Waltham, MA, USA) AlexaFlour 488 goat anti-mouse IgG secondary antibody (1:1000 dilution) for 30 min at RT with protection from light. The cells were counterstained with NucBlue Live Cell Stain (Waltham, MA, USA). The coverslips were mounted onto microscope slides using ProLong Diamond antifade medium (Waltham, MA, USA) and stored in the dark before imaging. Images were taken on a Zeiss LSM 980 with Airyscan 2 at 40× magnification. The images were analyzed using Adobe Photoshop 2023 software.

### 2.4. Mouse Immunizations

**Figure 2:**
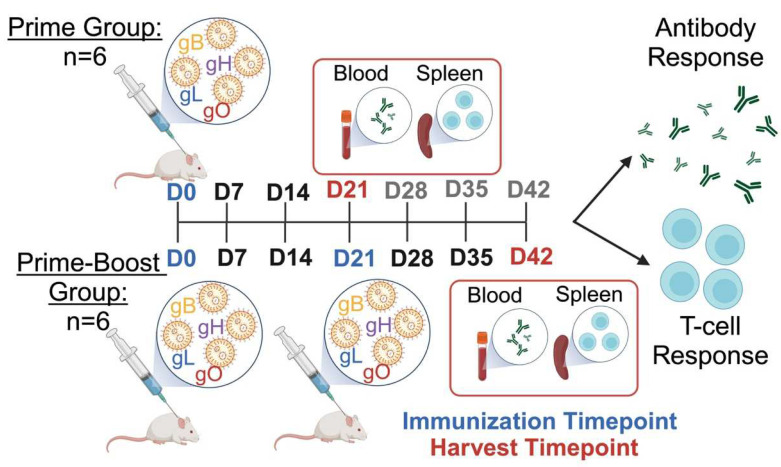
Experimental design of mouse study. Mice received either one or two injections of the EEHV1A mRNA + LNP vaccine or the luciferase mRNA + LNP vaccine. Mouse sera and spleens were harvested 3 weeks (D21) or 6 weeks (D42) post-injection(s) to measure antibody and T-cell responses. This diagram was created using BioRender.com.

There are no known established animal model systems for testing drugs or vaccines in elephants. However, prior to administering an expensive vaccine into a rare and endangered species, we wanted to confirm that it was immunogenic in an animal and had no adverse side effects. Mice were chosen as an affordable and convenient system to provide an assessment of these goals. CD-1 mice at 6–8 weeks of age purchased from the Center for Comparative Medicine at Baylor College of Medicine (Houston, TX, USA) were used in the vaccine study design shown in Figure 2. The outbred CD-1 mouse model was chosen to mirror the genetic diversity seen in elephants. Briefly, the mice were separated into prime (n = 6) and prime-boost (n = 6) groups. The prime group received one intramuscular injection of 10 μg mRNA + LNPs to a volume of 50 μL. After three weeks, whole spleens and blood were collected from the prime group for a downstream analysis. The prime-boost group received a booster vaccine injection of 10 μg mRNA + LNPs to a volume of 50 μL three weeks after the prime injection. The mice were euthanized three weeks after the booster, and samples were collected as described for the prime group. All of the mouse studies were approved by the Baylor College of Medicine Institutional Animal Care and Use Committee (IACUC).

### 2.5. The Immunoassay to Evaluate the EEHV1A-Specific Antibody Response

We used a luciferase immunoprecipitation (LIPS) assay to measure antibodies against gB, gH, gL, gO, FLAG, HA, and ORF-Q, as described previously [14,27,28,42]. Briefly, codon-optimized EEHV1A Kimba strain DNA sequences (GenBank accession: KC618527.1) for gB, gH, gL, gO, FLAG, HA, and ORF-Q were synthesized by GenScript (Piscataway, NJ, USA) and subcloned into the pGaus3 expression plasmid to generate Gaussia luciferase (GLuc)–antigen fusion proteins. Expression plasmids were transfected into the HEK293T cells, and supernatants and cell extracts were harvested and stored at −80 °C. The luciferase activity for each of the GLuc–antigen fusion proteins was determined using a Promega Glomax luminometer (Madison, WI, USA). The serum samples for the LIPS assay were diluted 1:10 in buffer A (50 mM Tris pH 7.5, 100 mM NaCl, 5 mM Mg Cl_2_, 1% Triton X-100) and stored at 4 °C for up to one month. We mixed 10 μL of diluted serum with 40 μL of buffer A (1:5 dilution). A master mix containing 1 × 10^7^ relative light units (RLU) per 50 μL of GLuc extract was made, and 50 μL of the GLuc extract master mix was added to the diluted samples in a 96-well polypropylene plate and incubated on a rotary shaker for 1 h at RT in the dark. The samples were run in duplicate. Following 1 h of incubation, 10 μL of a 30% GenDEPOTprotein A-G bead suspension (Katy, TX, USA) diluted in buffer A and the serum-GLuc mixture were added to a 1.2 μm hydrophilic Durapore membrane with low protein binding in a Millipore Sigma (Burlington, MA, USA) 96-well plate. The samples were incubated with constant shaking in the dark for 1 h at RT. The wells were washed with buffer A, followed by 1× PBS, using a Millipore Sigma 96-well vacuum manifold (Burlington, MA, USA). A BMG LAbtech OmniStar automatic plate luminometer (Ortenberg, Germany, EU) was used to inject 50 μL of the Promega Renilla luciferase assay substrate (Madison, WI, USA) into each well, with shaking for 2 s, followed by the recording of the RLU values for 5 s. The average RLU over 5 s post-injection was collected. GraphPad Prism 10 was used for the data analysis.

### 2.6. The Immunoassay to Evaluate EEHV1A-Specific T-Cell Response

The T-cell responses to gB, gH, gL, and gO in the vaccinated mice were measured for the prime (day 21) and prime-boost groups (day 42) as described previously [27,28,29]. We observed the greatest T-cell response in the mice that received the prime-boost dose (day 42). Therefore, only data for the prime-boost group (day 42) are shown. Briefly, mouse spleens were trimmed of excess fat and processed through a 70 μm filter. We added Biolegend 1× red blood cell lysis buffer (San Diego, CA, USA) to the homogenate and incubated at 4 °C for 3–5 min. Following red blood cell lysis, the splenocytes were centrifuged at 350× *g* for 5 min and resuspended in Lonza complete RPMI-1640 media (Walkersville, MA, USA) supplemented with 10% Gibco fetal bovine serum (FBS) (Waltham, MA, USA) and 1× Gibco penicillin/streptomycin (Waltham, MA, USA). After isolation, 2 × 10^6^ splenocytes were plated onto a 96-well round-bottom plate and stimulated at 37 °C overnight with either EEHV1A gB, gH, gL, or gO peptide pools or dimethyl sulfoxide (DMSO) at 1 μg/mL in complete RPMI-1640 medium and a Thermo Fisher Scientific (Waltham, MA, USA) 1× protein transport inhibitor cocktail. The EEHV1A gB, gH, gL (Mimotopes, Mulgrave, Australia), and gO (GeneMed Synthesis, San Antonio, TX, USA) peptides were synthesized and purified at 1 mg per vial. The peptide libraries consist of consecutive 15-mers overlapping by 11 amino acids spanning the length of gB (210 peptides), gH (182 peptides), gL (77 peptides), and gO (51 peptides). To prevent possible T-cell exhaustion and optimize the peptide concentrations, the gB and gH peptide libraries were separated into three different pools containing 70 peptides or 60–61 peptides each, respectively. Each peptide pool was mixed into DMSO at 10 mg/mL [25]. After overnight stimulation, the cells were washed and incubated with Fc Block (BD Biosciences, Franklin Lakes, NJ, USA) for 15 min at 4 °C. After blocking, the splenocytes were stained with extracellular antibodies for CD4 (BD Biosciences, Franklin Lakes, NJ, USA) (Clone GK1.5, 1:400 dilution), CD3 (Biolegend, San Diego, CA, USA) (Clone 17A2, 1:40 dilution), CD8a (Tonbo Biosciences, San Diego, CA, USA) Clone 53-6.7, 1:800 dilution), and Ghost Dye 780 viability dye (Tonbo Sciences, San Diego, CA, USA) (1:50 dilution) in Brilliant Stain Buffer (BD Sciences, Franklin Lakes, NJ, USA) at 4 °C for 30 min in the dark. The cells were fixed and permeabilized with Cytofix/Cytoperm solution (BD Biosciences, USA) for 20 min at 4 °C and washed with 1× perm/wash solution (BD Biosciences, Franklin Lakes, NJ, USA). After fixation and permeabilization, the cells were stained with intracellular cytokine antibodies for IFNγ (BD Biosciences, San Diego, CA, USA) (Clone XMG1.2, 1:50 dilution), TNFα (Biolegend, San Diego, CA, USA) (Clone MP6-XT22,1:40 dilution), and IL-4 (Biolegend, San Diego, CA, USA) (Clone 11B11, 1:25 dilution) diluted in a 1:5 mixture of 10× perm/wash buffer and Brilliant Stain Buffer (BD Biosciences, Franklin Lakes, NJ, USA) at 4 °C for 30 min in the dark. The cells were washed in 1× perm/wash buffer and resuspended in a buffer containing GenDEPOT Dulbecco’s PBS (Katy, TX, USA) and 2% Gibco FBS (Waltham, MA, USA) for analysis. An LRSII flow cytometer (BD Biosciences, Franklin Lakes, NJ, USA) was used to collect all of the data, and the analysis was performed using FlowJo software version 10.10 (BD Biosciences, Franklin Lakes, NJ, USA). CD4^+^ and CD8^+^ T cells were gated from the total CD3^+^ T-cell population. Dead cells were excluded from the analysis, and the background responses in the negative controls were subtracted from stimulated samples.

### 2.7. Statistical Analysis

Statistical analysis was carried out using GraphPad Prism 10. The LIPS data for the experimental and control groups are presented as geometric means ± the standard deviation (SD) of log10 values. The Shapiro–Wilk test was used to determine the normality of the data. One-way ANOVA with Sidak’s multiple comparison test was used to determine the significance of the normally distributed data, whereas the Kruskal–Wallis test was used for non-normally distributed data. A *p*-value ≤ 0.05 denoted statistical significance. The cut-off for determining the sensitivity and specificity for each viral antigen was derived from the mean antibody titer of the no-serum control samples plus 5 SDs. For the flow cytometry assays, the results are presented as the mean ± SD of the percentage of positive cells. The data are presented as the sum of the percentage of positive cells from all of the gB, gH, gL, and gO peptide pools per mouse. The Shapiro–Wilk test was used to determine the normality of the data. Outliers were identified using ROUT analysis (1%) and removed. The significance of the normally distributed data was determined through one-way ANOVA with Sidak’s multiple comparison test. Non-normally distributed data were analyzed using the Kruskal–Wallis test with Dunn’s correction. A *p*-value ≤ 0.05 denoted statistical significance.

## 3. Results

### 3.1. Protein Expression and Localization of Glycoproteins gB, gH, gL, and gO in the Transfected Cells

**Figure 3:**
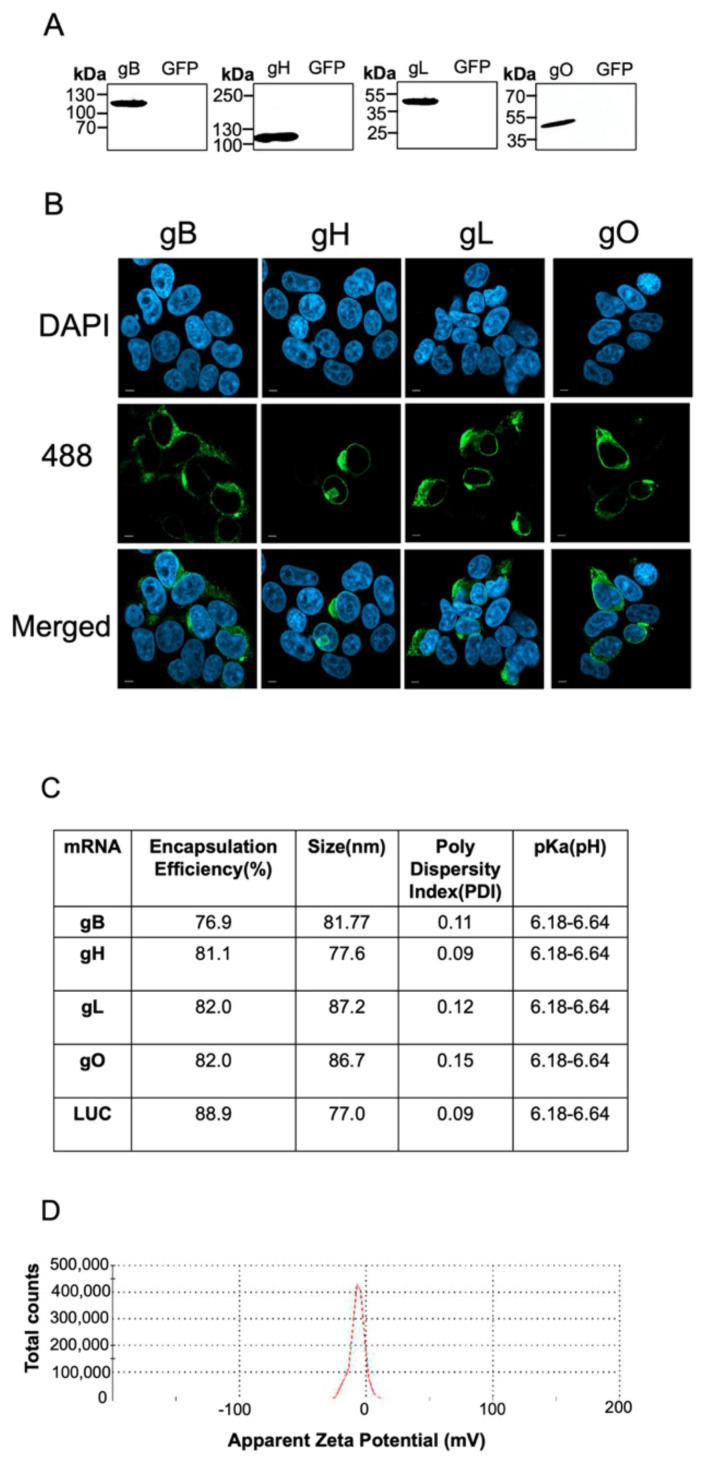
Characterization of mRNA translation in vitro and mRNA + LNP vaccine formulation. (**A**) Immunoblots of modified EEHV1A proteins expressed after mRNA transfection into HEK293T cells with the following protein sizes (kDa): gB (98.24 *), gH (86.84 *), gL (31.73 *), and gO (25.62 *). Asterisk (*) denotes predicted molecular weight does not include N– or O–linked glycosylation. Images were taken on an Azure Biosystems 300Q Chemiluminescent imager (Dublin, CA, USA)(**B**) Cells were imaged on a Zeiss LSM 980 Confocal Microscope with Airyscan 2 at 40× magnification and stained with NucBlue Live Cell stain (Hoechst 33342) and a secondary antibody conjugated to Alexa-488 to detect either HA or FLAG. A 10 µm scale bar is included in each image. (**C**) Encapsulation efficiency (%), particle size (nm), and pKa (pH) were determined using a DLS reader, a RiboGreen assay, and a TNS fluorescence assay, respectively. (**D**) A representative graph of the zeta potential distribution for all formulations is provided.

We evaluated the protein expression and localization in HEK293T cells transiently infected with EEHV1A gB, gH, gL, or gO mRNAs. The protein expression was measured through Western blot and immunofluorescence staining using antibodies against HA or FLAG epitopes. All of the glycoproteins migrated at higher than their predicted molecular weights, likely because of their predicted N- and O-linked glycosylation [43] (Figure 3A). The immunofluorescence staining indicated largely cytoplasmic localization, with some possible evidence of perinuclear localization, as seen for homologous proteins from other herpesviruses [19,44] (Figure 3B). Based on these positive expression data, we proceeded with packaging the mRNA into lipid nanoparticles (LNPs) and carried out quality control measures for the vaccine. The encapsulation efficiency for the various LNPs ranged from 76 to 89%, with the particle sizes averaging between 77 and 88 nm. The polydispersity index (PDI) was less than 0.20 for all formulations (Figure 3C). All of the vaccine formulations had particles with an average charge of −12.15 + −2.03 mV, as calculated from three separate measurements and as shown by the representative gO + LNP zeta potential graph (Figure 3D).

### 3.2. Multi-Antigen EEHV1A mRNA Vaccine Elicits Robust Antibody Responses in Mice

**Figure 4:**
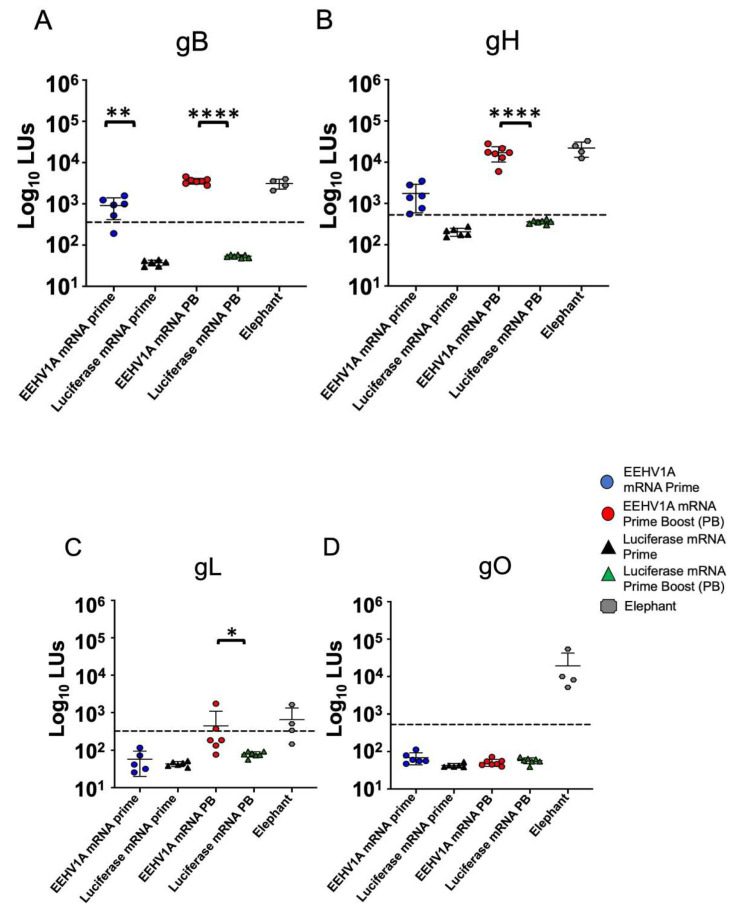
Multi-antigen mRNA vaccine induces antibodies in mice. (**A**) gB, (**B**) gH, (**C**) gL, and (**D**) gO antibodies in prime (D21) and prime-boost (D42) vaccine groups. Four elephants with previous EEHV1A infection were included in all of the LIPS assays as positive controls. Geometric means ± standard deviations (SDs) of log10 values are shown for each animal with two replicates. The limit of detection as shown by the dotted line is the mean plus 5 standard deviations of the negative control. Asterisks indicate statistical differences determined using one-way ANOVA with Sidak’s multiple comparison test or the Kruskal–Wallis test (* *p* ≤ 0.05) (** *p* ≤ 0.01) (**** *p* ≤ 0.0001).

**Figure 5:**
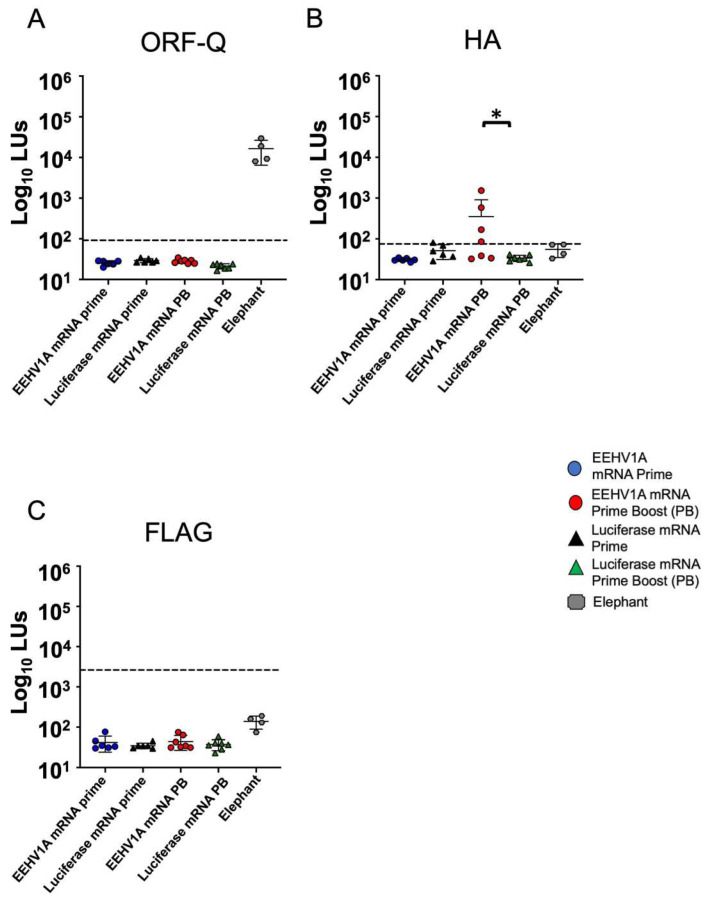
The multi-antigen mRNA vaccine induces variable levels of antibodies against HA and FLAG and no antibodies against ORF-Q in mice. (**A**) ORF-Q, (**B**) HA, and (**C**) FLAG antibodies in mice vaccinated with a prime and prime-boost EEHV1A + LNP vaccine. Four elephants with previous EEHV1A infection were included in all of the LIPS assays as positive controls. Geometric means ± SDs of log10 values are shown for each animal with two replicates. The limit of detection as shown by the dotted line is the mean plus 5 standard deviations of the negative control. Asterisks indicate statistical differences determined using one-way ANOVA with Sidak’s multiple comparison test or the Kruskal–Wallis test (* *p* ≤ 0.05).

We immunized CD-1 mice with an EEHV1A multi-antigen gB, gH, gL, and gO mRNA vaccine with a single (prime) or booster (prime-boost) dose. In the mice injected intramuscularly with a vaccine comprising 10 μg mRNA + LNPs, with a total of 2.5 μg per antigen, we observed no adverse effects following a single or booster dose of the EEHV1A mRNA + LNP vaccine. We measured the relative IgG titers with the LIPS assay using serum samples collected three weeks after either the prime or prime-boost dose. A significant antibody response to gB was observed three weeks after the prime dose (Figure 4A). Antibody titers against gB and gH increased after a booster dose to about 50-fold and 60-fold higher than those in the luciferase mRNA + LNP-vaccinated mice, respectively (Figure 4A,B). The antibody titers against gL in the EEHV1A mRNA + LNP-boosted mice increased 6-fold compared to those in the luciferase mRNA + LNP mice (Figure 4C); however, there was no response to gO in either the prime or boosted mice (Figure 4D). Compared with the serum titers for four adult elephants with a prior EEHV infection [14], a booster dose in the mice elicited similar average antibody titers for gB, gH, and gL (Figure 4A–C). However, the sensitivity of the LIPS assay may vary between species due to the antibody binding affinity to the protein A-G beads. There were no detectable antibodies against ORF-Q, an EEHV1A protein not included in the vaccine, in the vaccinated mice vs. naturally infected elephants (Figure 5A), indicating the specificity of the response. We observed variable HA antibody titers but no antibodies against FLAG in the EEHV1A mRNA + LNP-boosted mice (Figure 5B,C). Thus, our gB, gH, gL, and gO mRNA vaccine elicited EEHV-specific antibodies following a prime and booster dose of the vaccine.

### 3.3. The EEHV1A gB, gH, gL, and gO mRNA Vaccine Elicits CD4^+^ and CD8^+^ T-Cell Responses in Mice

**Figure 6:**
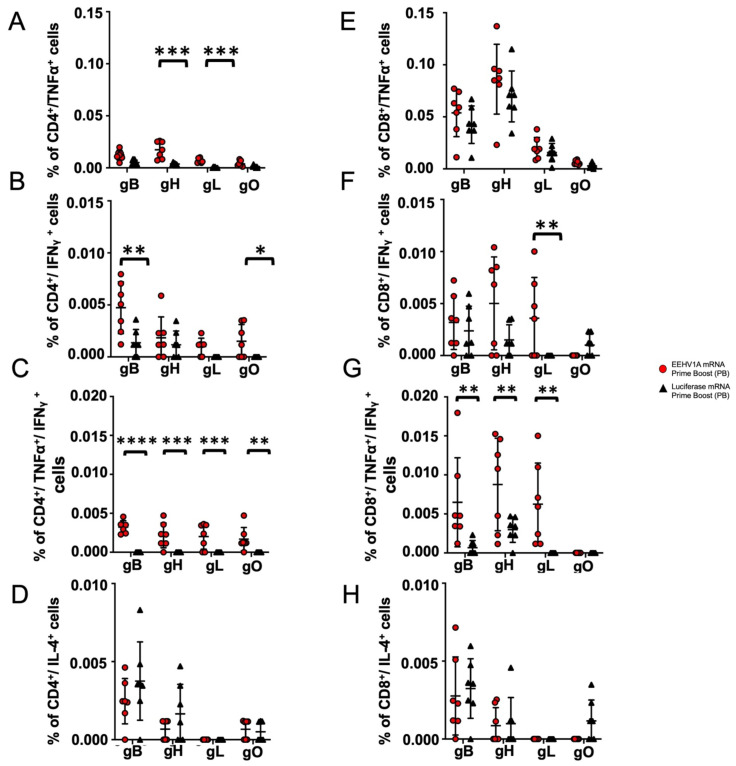
The multi-antigen mRNA vaccine induces a CD4^+^ and CD8^+^ T-cell response in mice following a booster dose. CD4^+^ and CD8^+^ splenocytes from immunized mice were assessed for IFNγ, TNFα, and IL-4 production following stimulation with gB, gH, gL, or gO peptide pools. Red circles indicate mice immunized with EEHV1A mRNA + LNPs, and black triangles represent mice immunized with luciferase mRNA + LNPs. (**A**) Percentage of CD4^+^ cells secreting TNFα following the prime-boost dose. (**B**) Percentage of CD4^+^ cells secreting IFNγ following the prime-boost dose. (**C**) Percentage of bi-functional CD4^+^ cells secreting both IFNγ and TNFα following the prime-boost dose. (**D**) Percentage of CD4^+^ cells secreting IL-4 following the prime dose. (**E**) Percentage of CD8^+^ cells secreting TNFα following the prime-boost dose. (**F**) Percentage of CD8^+^ cells secreting IFNγ following the prime-boost boost. (**G**) Percentage of bi-functional CD8^+^ cells secreting both IFNγ and TNFα following the prime-boost dose. (**H**) Percentage of CD8^+^ cells secreting IL-4 following the prime-boost dose. Mean ± SD values for each group are shown. Asterisks indicate statistical differences determined using one-way ANOVA with Sidak’s multiple comparison test or the Kruskal–Wallis test with Dunn’s correction for non-normally distributed data (* *p* ≤ 0.05) (** *p* ≤ 0.01) (*** *p* ≤ 0.001) (**** *p* ≤ 0.0001).

To measure the cell-mediated immune responses in the vaccinated CD-1 mice, we identified CD4^+^ and CD8^+^ cells secreting IFNγ, TNFα, and IL-4 after stimulation with the gB, gH, gL, or gO peptide pools using intracellular cytokine staining and flow cytometry. The highest responses were in the mice receiving the booster dose of the EEHV1A mRNA + LNP vaccine (Figure 6). We identified single and bi-functional CD4^+^ and CD8^+^ T cells reactive against gB, gH, gL, and gO vs. the DMSO controls. For gH and gL, a higher percentage of CD4^+^ cells secreted only TNFα vs. that in the control (Figure 6A), and for gB and gO, a higher percentage of CD4^+^ cells secreted only IFNᵧ (Figure 6B). However, for gB, gH, gL, and gO, some bi-functional CD4^+^ cells secreted both TNFα and IFNᵧ (Figure 6C). We determined that the vaccine did not induce a T_H_2 response according to the insignificant levels of CD4^+^ cells secreting IL-4 following a booster vaccine dose (Figure 6D). Although the CD8^+^ T-cell responses were less robust than those for CD4^+^, for gL, we observed a higher percentage of CD8^+^ cells secreting only IFNᵧ vs. the control (Figure 6F), and for gB, gH, and gL, a higher percentage of CD8^+^ cells secreted both TNFα and IFNᵧ (Figure 6G). There was also no significant percentage of CD8^+^ cells secreting IL-4 following a booster dose vs. the control (Figure 6H). Thus, our multi-antigen mRNA vaccine induced a T_H_1-specific T-cell response in mice that consisted mainly of bi-functional CD4^+^ T cells.

## 4. Discussion

We demonstrated here that a multi-antigen EEHV1A mRNA vaccine encoding gB, gH, gL, and gO encapsulated into LNPs induced an antibody and cellular immune response in mice with no adverse effects. Our multi-antigenic EEHV1A mRNA vaccine elicited high titers of EEHV-specific antibodies and bi-functional CD4^+^ and CD8^+^ T cells, with antibody levels, given the limitations of the LIPs assay, that are comparable to those in naturally infected adult elephants. Lethal EEHV-HD in young elephants is linked to a reduction in or loss of EEHV-reactive maternal antibodies preceding a primary infection [10,14,16]. Therefore, in young elephants, our vaccine should stimulate humoral and cellular immune responses against conserved herpesvirus proteins responsible for viral attachment and entry, which we hypothesize will protect them from lethal EEHV1A disease.

The activation of CD4^+^ and CD8^+^ cells in response to all four glycoproteins was weaker than the responses we saw in our prior studies after vaccination with recombinant MVA or adjuvanted subunit vaccines [27,28,29]. For both vaccines, we demonstrated a dominant bi-functional CD4^+^ T-cell response to the gH/gL complex and a bi-functional CD4^+^ and CD8^+^ T-cell response to gB [27,28,29]. Although the mRNA vaccine induced a CD4^+^-dominant T-cell response like the adjuvanted gH/gL subunit vaccine, the overall T-cell response was less than that for either the adjuvanted subunit vaccine or the recombinant MVA vaccine. The T-cell response in the adjuvanted gB and gH/gL subunit vaccines may have been due to the saponin and the Toll-like receptor ligand-based adjuvant used in the formulation [45]. Although the protection against lethal disease in young elephants that can be provided by the dominant CD4^+^ response is unknown, cytotoxic CD4^+^ cells also play a role in anti-herpesvirus immunity [46,47].

Glycoprotein gO, which forms a complex with gH/gL, is thought to be the component that interacts with the cellular receptor for viral entry [48]. However, although gB, gH, and gL antibody titers increased following a boost, there were no detectable antibodies against gO. In contrast, three 50 μg doses of a gO DNA vaccine for HCMV induce neutralizing antibodies against gO in inbred mice [49]. However, we deliberately chose the outbred CD-1 mouse model to mirror the genetic diversity in elephants. The differences associated with various vaccine platforms are not well studied; however, inbred and outbred strains vaccinated with multi-antigenic peptides and an adjuvant show differences in antibody and T-cell responses [50]. A dose of 2.5 μg of gO mRNA induced a CD4^+^ T-cell response, suggesting that this glycoprotein is expressed after vaccination. However, as vaccine efficacy is influenced by host genetics [51], our lack of an antibody response to gO could be genetic, suggesting that these results in mice may not be transferable to elephants. Alternatively, gO being expressed in isolation or in murine cells instead of elephant cells may alter its intrinsic immunogenicity. Regardless, preliminary evidence emerging in a small trial vaccination of elephants using the same mRNA vaccine used in our mouse studies is showing the induction of anti-gO antibody responses (unpublished observations).

Because there are no EEHV in vitro culture systems, there is no assay for detecting EEHV-neutralizing antibodies. However, in response to vaccines for other herpesviruses, such as HCMV and Kaposi’s sarcoma-associated herpesvirus, and in naturally infected individuals, there are neutralizing antibodies against the gH/gL complex and gB [52,53]. An EBV gH/gL nanoparticle vaccine protected humanized mice from a viral challenge [54], and non-neutralizing antibodies against gB have protected against HSV virus and HCMV infections [55,56]. Thus, these previous studies suggest that our mRNA vaccine likely induced neutralizing and non-neutralizing antibodies that mediated antibody-dependent cell-mediated phagocytosis. We demonstrated that elephants that succumbed to infection are seronegative for the EEHV type that caused the disease [10,14], indicating the importance of antibodies in preventing EEHV-HD.

At present, our approach has two limitations: (1) the mRNAs for each glycoprotein are packaged separately into LNPs that are then mixed together, and (2) our LIPS assays only detect the trimeric gH/gL/gO glycoprotein antigens individually. Thus, the current vaccine formulation may preclude the abundant expression of all glycoproteins in a single cell in vivo, and formation of the trimeric complex of gH/gL/gO may be limited. While it is unknown whether conformation-specific antibodies against the trimeric complex are critical for neutralization or protection, it may still be beneficial to modify our vaccine design to increase the probability that all three glycoproteins are delivered and expressed in the same cell so that these potential complexes are formed. We are currently modifying our LIPS assays to express the gH/gL/gO trimer for the detection of antibodies against the complex.

The durability of mRNA vaccine response, particularly for the COVID-19 mRNA vaccines, has been measured [57,58,59,60]. Although we demonstrated significant humoral and cell-mediated immune responses three weeks after a booster dose, we do not know how long the response will endure. Further, heterologous vaccines may be more immunogenic and elicit a longer-lasting humoral and cell-mediated immune response than homologous vaccines [61]. We are now assessing the immunogenicity of a heterologous prime-boost with our multi-antigen mRNA vaccine and an adjuvanted protein subunit vaccine that includes gB and the gH/gL/gO trimer complex. Additionally, there is an ongoing vaccination trial in eight elephants to evaluate the immunogenicity of our mRNA vaccine in Asian elephants and evaluate the durability of their immune response over six months. Taken together, these studies will be important for determining the most robust vaccination regimen for reducing lethal HD in young Asian elephants.

## 5. Conclusions

In summary, for the first time, we generated a multi-antigen EEHV1A mRNA vaccine that induced strong, specific antibody and T-cell responses in outbred mice. This EEHV1A mRNA vaccine, incorporating gB, gH, gL, and gO, elicited a robust EEHV-specific immune response in mice and provides the foundation for future trials in Asian elephants.

## Data Availability

Raw data files for the results presented in this work are available upon request.

## References

[1] Long S.Y., Latimer E.M., Hayward G.S. (2016). Review of Elephant Endotheliotropic Herpesviruses and Acute Hemorrhagic Disease. Ilar J..

[2] Fayette M.A., Brenner E.E., Garner M.M., Bowman M.R., Latimer E., Proudfoot J.S. (2021). Acute Hemorrhagic Disease Due to Elephant Endotheliotropic Herpesvirus 3a Infection in Five African Elephants (*Loxodonta africana*) at One North American Zoological Institution. J. Zoo. Wildl. Med..

[3] Richman L.K., Montali R.J., Garber R.L., Kennedy M.A., Lehnhardt J., Hildebrandt T., Schmitt D., Hardy D., Alcendor D.J., Hayward G.S. (1999). Novel endotheliotropic herpesviruses fatal for Asian and African elephants. Science.

[4] Zong J.C., Heaggans S.Y., Long S.Y., Latimer E.M., Nofs S.A., Bronson E., Casares M., Fouraker M.D., Pearson V.R., Richman L.K. (2015). Detection of Quiescent Infections with Multiple Elephant Endotheliotropic Herpesviruses (EEHVs), Including EEHV2, EEHV3, EEHV6, and EEHV7, within Lymphoid Lung Nodules or Lung and Spleen Tissue Samples from Five Asymptomatic Adult African Elephants. J. Virol..

[5] Bronson E., McClure M., Sohl J., Wiedner E., Cox S., Latimer E.M., Pearson V.R., Hayward G.S., Fuery A., Ling P.D. (2017). Epidemiologic Evaluation of Elephant Endotheliotropic Herpesvirus 3b Infection in an African Elephant (*Loxodonta africana*). J. Zoo Wildl. Med..

[6] Zong J.C., Latimer E.M., Long S.Y., Richman L.K., Heaggans S.Y., Hayward G.S. (2014). Comparative genome analysis of four elephant endotheliotropic herpesviruses, EEHV3, EEHV4, EEHV5, and EEHV6, from cases of hemorrhagic disease or viremia. J. Virol..

[7] Richman L.K., Zong J.C., Latimer E.M., Lock J., Fleischer R.C., Heaggans S.Y., Hayward G.S. (2014). Elephant endotheliotropic herpesviruses EEHV1A, EEHV1B, and EEHV2 from cases of hemorrhagic disease are highly diverged from other mammalian herpesviruses and may form a new subfamily. J. Virol..

[8] Stanton J.J., Nofs S.A., Peng R., Hayward G.S., Ling P.D. (2012). Development and validation of quantitative real-time polymerase chain reaction assays to detect elephant endotheliotropic herpesviruses-2, 3, 4, 5, and 6. J. Virol. Methods.

[9] Stanton J.J., Nofs S.A., Zachariah A., Kalaivannan N., Ling P.D. (2014). Detection of elephant endotheliotropic herpesvirus infection among healthy Asian elephants (*Elephas maximus*) in South India. J. Wildl. Dis..

[10] Pursell T., Spencer Clinton J.L., Tan J., Peng R., Qin X., Doddapaneni H., Menon V., Momin Z., Kottapalli K., Howard L. (2021). Primary Infection May Be an Underlying Factor Contributing to Lethal Hemorrhagic Disease Caused by Elephant Endotheliotropic Herpesvirus 3 in African Elephants (*Loxodonta africana*). Microbiol. Spectr..

[11] Richman L.K., Montali R.J., Cambre R.C., Schmitt D., Hardy D., Hildbrandt T., Bengis R.G., Hamzeh F.M., Shahkolahi A., Hayward G.S. (2000). Clinical and pathological findings of a newly recognized disease of elephants caused by endotheliotropic herpesviruses. J. Wildl. Dis..

[12] Khammesri S., Mathura Y., Boonprasert K., Ampasavate C., Hongwiset D., Brown J.L., Thitaram C. (2021). Successful treatment of elephant endotheliotropic herpesvirus infection in an Asian elephant (*Elephas maximus*) calf by oral acyclovir medication: Case report. J. Vet. Med. Sci..

[13] Dastjerdi A., Seilern-Moy K., Darpel K., Steinbach F., Molenaar F. (2016). Surviving and fatal Elephant Endotheliotropic Herpesvirus-1A infections in juvenile Asian elephants—Lessons learned and recommendations on anti-herpesviral therapy. BMC Vet. Res..

[14] Fuery A., Pursell T., Tan J., Peng R., Burbelo P.D., Hayward G.S., Ling P.D. (2020). Lethal Hemorrhagic Disease and Clinical Illness Associated with Elephant Endotheliotropic Herpesvirus 1 Are Caused by Primary Infection: Implications for the Detection of Diagnostic Proteins. J. Virol..

[15] Nofs S.A., Atmar R.L., Keitel W.A., Hanlon C., Stanton J.J., Tan J., Flanagan J.P., Howard L., Ling P.D. (2013). Prenatal passive transfer of maternal immunity in Asian elephants (*Elephas maximus*). Vet. Immunol. Immunopathol..

[16] Hoornweg T.E., Perera V.P., Karunarathne R.N.S., Schaftenaar W., Mahakapuge T.A.N., Kalupahana A.W., Rutten V., de Haan C.A.M. (2022). Young elephants in a large herd maintain high levels of elephant endotheliotropic herpesvirus-specific antibodies and do not succumb to fatal haemorrhagic disease. Transbound. Emerg. Dis..

[17] Connolly S.A., Jackson J.O., Jardetzky T.S., Longnecker R. (2011). Fusing structure and function: A structural view of the herpesvirus entry machinery. Nat. Rev. Microbiol..

[18] Harnois M.J., Dennis M., Stohr D., Valencia S.M., Rodgers N., Semmes E.C., Webster H.S., Jenks J.A., Barfield R., Pollara J. (2022). Characterization of Plasma Immunoglobulin G Responses in Elite Neutralizers of Human Cytomegalovirus. J. Infect. Dis..

[19] Ciferri C., Chandramouli S., Donnarumma D., Nikitin P.A., Cianfrocco M.A., Gerrein R., Feire A.L., Barnett S.W., Lilja A.E., Rappuoli R. (2015). Structural and biochemical studies of HCMV gH/gL/gO and Pentamer reveal mutually exclusive cell entry complexes. Proc. Natl. Acad. Sci. USA.

[20] Wang B., Hara K., Kawabata A., Nishimura M., Wakata A., Tjan L.H., Poetranto A.L., Yamamoto C., Haseda Y., Aoshi T. (2020). Tetrameric glycoprotein complex gH/gL/gQ1/gQ2 is a promising vaccine candidate for human herpesvirus 6B. PLoS Pathog..

[21] Zhong L., Zhang W., Krummenacher C., Chen Y., Zheng Q., Zhao Q., Zeng M.S., Xia N., Zeng Y.X., Xu M. (2023). Targeting herpesvirus entry complex and fusogen glycoproteins with prophylactic and therapeutic agents. Trends Microbiol..

[22] Awasthi S., Knox J.J., Desmond A., Alameh M.G., Gaudette B.T., Lubinski J.M., Naughton A., Hook L.M., Egan K.P., Tam Y.K. (2021). Trivalent nucleoside-modified mRNA vaccine yields durable memory B cell protection against genital herpes in preclinical models. J. Clin. Investig..

[23] Ling P.D., Long S.Y., Fuery A., Peng R.S., Heaggans S.Y., Qin X., Worley K.C., Dugan S., Hayward G.S. (2016). Complete Genome Sequence of Elephant Endotheliotropic Herpesvirus 4, the First Example of a GC-Rich Branch Proboscivirus. mSphere.

[24] Hoornweg T.E., Schaftenaar W., Maurer G., van den Doel P.B., Molenaar F.M., Chamouard-Galante A., Vercammen F., Rutten V., de Haan C.A.M. (2021). Elephant Endotheliotropic Herpesvirus Is Omnipresent in Elephants in European Zoos and an Asian Elephant Range Country. Viruses.

[25] Fuery A., Leen A.M., Peng R., Wong M.C., Liu H., Ling P.D. (2018). Asian Elephant T Cell Responses to Elephant Endotheliotropic Herpesvirus. J. Virol..

[26] Pavulraj S., Eschke K., Prahl A., Flugger M., Trimpert J., van den Doel P.B., Andreotti S., Kaessmeyer S., Osterrieder N., Azab W. (2019). Fatal Elephant Endotheliotropic Herpesvirus Infection of Two Young Asian Elephants. Microorganisms.

[27] Pursell T., Spencer Clinton J.L., Tan J., Peng R., Ling P.D. (2022). Modified vaccinia Ankara expressing EEHV1A glycoprotein B elicits humoral and cell-mediated immune responses in mice. PLoS ONE.

[28] Spencer Clinton J.L., Hoornweg T.E., Tan J., Peng R., Schaftenaar W., Rutten V., de Haan C.A.M., Ling P.D. (2022). EEHV1A glycoprotein B subunit vaccine elicits humoral and cell-mediated immune responses in mice. Vaccine.

[29] Spencer Clinton J.L., Hoornweg T.E., Tan J., Peng R., Schaftenaar W., Rutten V., de Haan C.A.M., Ling P.D. (2024). The EEHV1A gH/gL complex elicits humoral and cell-mediated immune responses in mice. Vaccine.

[30] Liu M.A. (2010). Immunologic basis of vaccine vectors. Immunity.

[31] Chen N., Xia P., Li S., Zhang T., Wang T.T., Zhu J. (2017). RNA sensors of the innate immune system and their detection of pathogens. IUBMB Life.

[32] John S., Yuzhakov O., Woods A., Deterling J., Hassett K., Shaw C.A., Ciaramella G. (2018). Multi-antigenic human cytomegalovirus mRNA vaccines that elicit potent humoral and cell-mediated immunity. Vaccine.

[33] Nelson C.S., Jenks J.A., Pardi N., Goodwin M., Roark H., Edwards W., McLellan J.S., Pollara J., Weissman D., Permar S.R. (2020). Human Cytomegalovirus Glycoprotein B Nucleoside-Modified mRNA Vaccine Elicits Antibody Responses with Greater Durability and Breadth than MF59-Adjuvanted gB Protein Immunization. J. Virol..

[34] Monslow M.A., Elbashir S., Sullivan N.L., Thiriot D.S., Ahl P., Smith J., Miller E., Cook J., Cosmi S., Thoryk E. (2020). Immunogenicity generated by mRNA vaccine encoding VZV gE antigen is comparable to adjuvanted subunit vaccine and better than live attenuated vaccine in nonhuman primates. Vaccine.

[35] Jiang Z., Zhu L., Cai Y., Yan J., Fan Y., Lv W., Gong S., Yin X., Yang X., Sun X. (2020). Immunogenicity and protective efficacy induced by an mRNA vaccine encoding gD antigen against pseudorabies virus infection. Vet. Microbiol..

[36] Awasthi S., Hook L.M., Pardi N., Wang F., Myles A., Cancro M.P., Cohen G.H., Weissman D., Friedman H.M. (2019). Nucleoside-modified mRNA encoding HSV-2 glycoproteins C, D, and E prevents clinical and subclinical genital herpes. Sci. Immunol..

[37] Wei C.J., Bu W., Nguyen L.A., Batchelor J.D., Kim J., Pittaluga S., Fuller J.R., Nguyen H., Chou T.H., Cohen J.I. (2022). A bivalent Epstein-Barr virus vaccine induces neutralizing antibodies that block infection and confer immunity in humanized mice. Sci. Transl. Med..

[38] Lv J., Meng S., Gu Q., Zheng R., Gao X., Kim J.D., Chen M., Xia B., Zuo Y., Zhu S. (2023). Epigenetic landscape reveals MECOM as an endothelial lineage regulator. Nat. Commun..

[39] Mojiri A., Walther B.K., Jiang C., Matrone G., Holgate R., Xu Q., Morales E., Wang G., Gu J., Wang R. (2021). Telomerase therapy reverses vascular senescence and extends lifespan in progeria mice. Eur. Heart J..

[40] Chen S., Tam Y.Y.C., Lin P.J.C., Sung M.M.H., Tam Y.K., Cullis P.R. (2016). Influence of particle size on the in vivo potency of lipid nanoparticle formulations of siRNA. J. Control Release.

[41] Legere R.M., Poveda C., Ott J.A., Bray J.M., Villafone E.G., Silveira B.P.D., Kahn S.K., Martin C.L., Mancino C., Taraballi F. (2024). Intramuscular but not nebulized administration of a mRNA vaccine against *Rhodococcus equi* stimulated humoral immune responses in neonatal foals. Am. J. Vet. Res..

[42] Burbelo P.D., Ching K.H., Klimavicz C.M., Iadarola M.J. (2009). Antibody profiling by Luciferase Immunoprecipitation Systems (LIPS). J. Vis. Exp..

[43] Gasteiger E., Gattiker A., Hoogland C., Ivanyi I., Appel R.D., Bairoch A. (2003). ExPASy: The proteomics server for in-depth protein knowledge and analysis. Nucleic Acids Res..

[44] Atanasiu D., Whitbeck J.C., Cairns T.M., Reilly B., Cohen G.H., Eisenberg R.J. (2007). Bimolecular complementation reveals that glycoproteins gB and gH/gL of herpes simplex virus interact with each other during cell fusion. Proc. Natl. Acad. Sci. USA.

[45] Wang P. (2021). Natural and Synthetic Saponins as Vaccine Adjuvants. Vaccines.

[46] Marshall N.B., Swain S.L. (2011). Cytotoxic CD4 T cells in antiviral immunity. J. Biomed. Biotechnol..

[47] Walton S., Mandaric S., Oxenius A. (2013). CD4 T cell responses in latent and chronic viral infections. Front. Immunol..

[48] Connolly S.A., Jardetzky T.S., Longnecker R. (2021). The structural basis of herpesvirus entry. Nat. Rev. Microbiol..

[49] Wang H., Huang C., Dong J., Yao Y., Xie Z., Liu X., Zhang W., Fang F., Chen Z. (2015). Complete protection of mice against lethal murine cytomegalovirus challenge by immunization with DNA vaccines encoding envelope glycoprotein complex III antigens gH, gL and gO. PLoS ONE.

[50] Wolf A.I., Mozdzanowska K., Williams K.L., Singer D., Richter M., Hoffmann R., Caton A.J., Otvos L., Erikson J. (2011). Vaccination with M2e-based multiple antigenic peptides: Characterization of the B cell response and protection efficacy in inbred and outbred mice. PLoS ONE.

[51] Cruz Cisneros M.C., Anderson E.J., Hampton B.K., Parotti B., Sarkar S., Taft-Benz S., Bell T.A., Blanchard M., Dillard J.A., Dinnon K.H. (2024). Host Genetic Variation Impacts SARS-CoV-2 Vaccination Response in the Diversity Outbred Mouse Population. Vaccines.

[52] Mortazavi Y., Lidenge S.J., Tran T., West J.T., Wood C., Tso F.Y. (2020). The Kaposi’s Sarcoma-Associated Herpesvirus (KSHV) gH/gL Complex Is the Predominant Neutralizing Antigenic Determinant in KSHV-Infected Individuals. Viruses.

[53] Fouts A.E., Chan P., Stephan J.P., Vandlen R., Feierbach B. (2012). Antibodies against the gH/gL/UL128/UL130/UL131 complex comprise the majority of the anti-cytomegalovirus (anti-CMV) neutralizing antibody response in CMV hyperimmune globulin. J. Virol..

[54] Malhi H., Homad L.J., Wan Y.H., Poudel B., Fiala B., Borst A.J., Wang J.Y., Walkey C., Price J., Wall A. (2022). Immunization with a self-assembling nanoparticle vaccine displaying EBV gH/gL protects humanized mice against lethal viral challenge. Cell Rep. Med..

[55] Kuraoka M., Aschner C.B., Windsor I.W., Mahant A.M., Garforth S.J., Kong S.L., Achkar J.M., Almo S.C., Kelsoe G., Herold B.C. (2023). A non-neutralizing glycoprotein B monoclonal antibody protects against herpes simplex virus disease in mice. J. Clin. Investig..

[56] Bootz A., Karbach A., Spindler J., Kropff B., Reuter N., Sticht H., Winkler T.H., Britt W.J., Mach M. (2017). Protective capacity of neutralizing and non-neutralizing antibodies against glycoprotein B of cytomegalovirus. PLoS Pathog..

[57] Arunachalam P.S., Lai L., Samaha H., Feng Y., Hu M., Hui H.S., Wali B., Ellis M., Davis-Gardner M.E., Huerta C. (2023). Durability of immune responses to mRNA booster vaccination against COVID-19. J. Clin. Investig..

[58] Widge A.T., Rouphael N.G., Jackson L.A., Anderson E.J., Roberts P.C., Makhene M., Chappell J.D., Denison M.R., Stevens L.J., Pruijssers A.J. (2021). Durability of Responses after SARS-CoV-2 mRNA-1273 Vaccination. N. Engl. J. Med..

[59] Haq M.A., Roy A.K., Ahmed R., Kuddusi R.U., Sinha M., Hossain M.S., Vandenent M., Islam M.Z., Zaman R.U., Kibria M.G. (2024). Antibody longevity and waning following COVID-19 vaccination in a 1-year longitudinal cohort in Bangladesh. Sci. Rep..

[60] Korosec C.S., Farhang-Sardroodi S., Dick D.W., Gholami S., Ghaemi M.S., Moyles I.R., Craig M., Ooi H.K., Heffernan J.M. (2022). Long-term durability of immune responses to the BNT162b2 and mRNA-1273 vaccines based on dosage, age and sex. Sci. Rep..

[61] Lu S. (2009). Heterologous prime-boost vaccination. Curr. Opin. Immunol..

