# Correction: Watts et al. Multi-Antigen Elephant Endotheliotropic Herpesvirus (EEHV) mRNA Vaccine Induces Humoral and Cell-Mediated Responses in Mice. *Vaccines* 2024, *12*, 1429

**DOI:** 10.3390/vaccines14050438

**Published:** 2026-05-14

**Authors:** Jessica R. Watts, Jennifer L. Spencer Clinton, Jeroen Pollet, Rongsheng Peng, Jie Tan, Paul D. Ling

**Affiliations:** 1 Department of Molecular Virology and Microbiology, Baylor College of Medicine, Houston, TX 77030, USA; jwatts@bcm.edu (J.R.W.); rpeng@bcm.edu (R.P.); jtan@bcm.edu (J.T.); 2 Department of Pediatrics, Division of Tropical Medicine, Baylor College of Medicine, Houston, TX 77030, USA; jennifer.clinton@bcm.edu (J.L.S.C.); jeroen.pollet@bcm.edu (J.P.); 3 National School of Tropical Medicine, Baylor College of Medicine, Houston, TX 77030, USA

The authors would like to make the following corrections to this published paper [1].

In the original publication, there was a mistake in Figure 1A as published. The current diagram in Figure 1A shows epitope tags HA and Flag after the 3’UTR. This is an error. The HA and Flag epitope tags should be shown contiguous with the end of each open reading frame. The corrected Figure 1A appears below.

The authors state that the scientific conclusions are unaffected. This correction was approved by the Academic Editor. The original publication has also been updated.

## Figures and Tables

**Figure 1:**
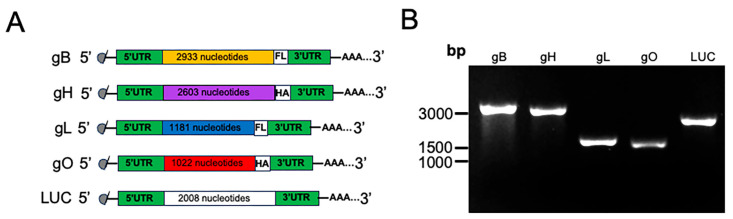
Characterization of in vitro-transcribed mRNA integrity. (**A**) Schematic of mRNA transcripts. Created in BioRender.com. Carboxy-terminal HA and FLAG(FL) protein epitope tags are indicated in the diagram. (**B**) mRNA transcripts visualized on a 1% native agarose gel. Luciferase mRNA (LUC) was included as the control for the vaccine study.
